# The Predictive Role of the Upper-Airway Adipose Tissue in the Pathogenesis of Obstructive Sleep Apnoea

**DOI:** 10.3390/life12101543

**Published:** 2022-10-04

**Authors:** Viktória Molnár, Zoltán Lakner, András Molnár, Dávid László Tárnoki, Ádám Domonkos Tárnoki, László Kunos, Zsófia Jokkel, László Tamás

**Affiliations:** 1 Department of Otolaryngology and Head and Neck Surgery, Semmelweis University, 1083 Budapest, Hungary; 2 Szent István Campus, Hungarian University of Agriculture and Life Sciences, 2100 Gödöllő, Hungary; 3 Medical Imaging Centre, Semmelweis University, 1082 Budapest, Hungary; 4 Institute of Pulmonology, Törökbálint, 2045 Törökbálint, Hungary; 5 Department of Voice, Speech and Swallowing Therapy, Faculty of Health Sciences, Semmelweis University, 1088 Budapest, Hungary

**Keywords:** obstructive sleep apnoea, MRI, obesity, parapharyngeal adipose tissue, artificial intelligence, drug-induced sleep endoscopy

## Abstract

**Simple Summary:**

Obstructive sleep apnoea (OSA) is an underdiagnosed disorder from which many patients are suffering, and may lead to severe complications. The adipose tissue near the upper airways is essential in upper-airway collapses and OSA severity. The present investigation aimed to determine the correlations between upper-airway adipose tissue MRI parameters and OSA, using artificial intelligence to analyse the pathophysiology of OSA and predict obstruction location. Including anthropometric and MRI adipose tissue parameters, OSA and upper-airway obstruction can be predicted with high precision. Artificial intelligence can effectively be used in OSA diagnostics as it can analyse non-linear correlations; thus, it can be helpful for undiagnosed OSA cases.

**Abstract:**

This study aimed to analyse the thickness of the adipose tissue (AT) around the upper airways with anthropometric parameters in the prediction and pathogenesis of OSA and obstruction of the upper airways using artificial intelligence. One hundred patients were enrolled in this prospective investigation, who were divided into control (non-OSA) and mild, moderately severe, and severe OSA according to polysomnography. All participants underwent drug-induced sleep endoscopy, anthropometric measurements, and neck MRI. The statistical analyses were based on artificial intelligence. The midsagittal SAT, the parapharyngeal fat, and the midsagittal tongue fat were significantly correlated with BMI; however, no correlation with AHI was observed. Upper-airway obstruction was correctly categorised in 80% in the case of the soft palate, including parapharyngeal AT, sex, and neck circumference parameters. Oropharyngeal obstruction was correctly predicted in 77% using BMI, parapharyngeal AT, and abdominal circumferences, while tongue-based obstruction was correctly predicted in 79% using BMI. OSA could be predicted with 99% precision using anthropometric parameters and AT values from the MRI. Age, neck circumference, midsagittal and parapharyngeal tongue fat values, and BMI were the most vital parameters in the prediction. Basic anthropometric parameters and AT values based on MRI are helpful in predicting OSA and obstruction location using artificial intelligence.

## 1. Introduction

Obstructive sleep apnoea is the most common sleep-related breathing disorder and, in unattended cases, is a major public health problem due to the background comorbidities [1]. Its increasing prevalence can be explained by the dynamic increase in obesity, the most crucial risk factor for OSA [2]. The prevalence of obesity has tripled since 1975. In 2016, 39% of adults were overweight and 13% obese, representing 1.9 billion and 650 million people, respectively [3]. Obesity is typical for developed countries, and explained by increased calorie intake, physical inactivity, and changes in the gut microbiome [4]. In addition to OSA, obesity is also a risk factor for other conditions, such as insulin resistance, diabetes mellitus, hypertension, atherosclerosis, stroke, or myocardial infarction [5]. Obesity can be classified into visceral and general types, of which visceral obesity is critical due to its decreasing effects on lung volumes and pharyngeal wall tension [6]. Although the pathophysiology of OSA is complex and multifactorial, impaired dilator muscle functions, ineffective loop gain, and low arousal threshold are its essential background [7]. Upper-airway obstruction can be the result of deposits of adipose tissue near the upper airways, which can be examined by CT or MRI. The significance of the parapharyngeal adipose corpus was first mentioned by Wlofram-Gabel et al., in 1996 [8]. The parapharyngeal region, the tongue, and subcutaneous adipose tissue of the neck lead to upper-airway obstruction in different ways. The correlations between OSA, obesity, and anthropometric parameters have been particularly investigated in several studies to analyse the pathophysiology of OSA in a more detailed manner and to predict OSA. Of the anthropometric parameters, BMI, neck, abdominal, and hip circumferences and waist–hip ratio are mainly investigated. In the recent ELSA-Brasil study, which included 2059 patients, all parameters mentioned above were found to be significantly higher in the OSA group than in the non-OSA group [9]. The Sleep Heart study, which included 6167 patients, observed significantly higher BMI and neck and hip circumferences in the case of severe OSA. However, it was also concluded that the BMI cut-off does not precisely represent the severity of obesity in different races and sexes [10]. The predictive role of anthropometric parameters in OSA depends on the sexes. In women, waist circumference and waist-to-height ratio were the most crucial parameters in predicting OSA, while in men, neck circumference and waist-to-height ratio were crucial in predicting mild OSA, and BMI in severe OSA [11]. The Wisconsin Sleep Cohort study, conducted in the USA, including 1520 participants between 30 and 70 years of age, observed a higher prevalence of sleep-related breathing disorders in older men with higher BMI values. Moreover, BMI was also the most strongly correlated with sleep-disordered breathing in younger participants [12].

The anthropometric parameters and the parameters of the adipose tissue near the upper airways can be analysed using medical imaging methods, resulting in large databases. The complex pathophysiology behind OSA cannot be described using simple statistical methods in all cases. Given the fast improvement in sciences, using artificial intelligence is advantageous in diagnostics, prediction, and therapy. Although many possibilities regarding OSA diagnostics are accessible (e.g., self-administered questionnaires, home sleep tests, or polysomnography), the ratio of undiagnosed cases is still high.

In the last two decades, the improvement in bioinformatics and artificial intelligence has allowed easy and rapid detection of OSA. At first, machine learning-based models included essential risk factors for OSA (i.e., age, sex, BMI, or neck circumference) [13,14], while others performed a prediction using anthropometric and faciocervical measurements [15]. Regarding the methods, the most vital expectations were simplicity and rapidity; therefore, ECG [16] and oxyhaemoglobin saturation [17], despite their effectiveness in prediction, cannot be integrated into daily practice. Other studies investigated the use of artificial intelligence to predict OSA using questionnaires [18]. The prediction of OSA was also successful by 2D imaging [19] and 3D face reconstruction using artificial intelligence [20].

In the present study, in addition to anthropometric parameters, the upper-airway adipose tissue was examined using MRI in an OSA population to analyse its effects on OSA pathogenesis. Furthermore, the prediction of OSA and upper-airway obstruction, including the parameters mentioned above, was also investigated using artificial intelligence (the Flexible Discriminance analysis and the Multivariate Adaptive Regression Splines).

## 2. Materials and Methods

### 2.1. Participants

**Figure 1:**
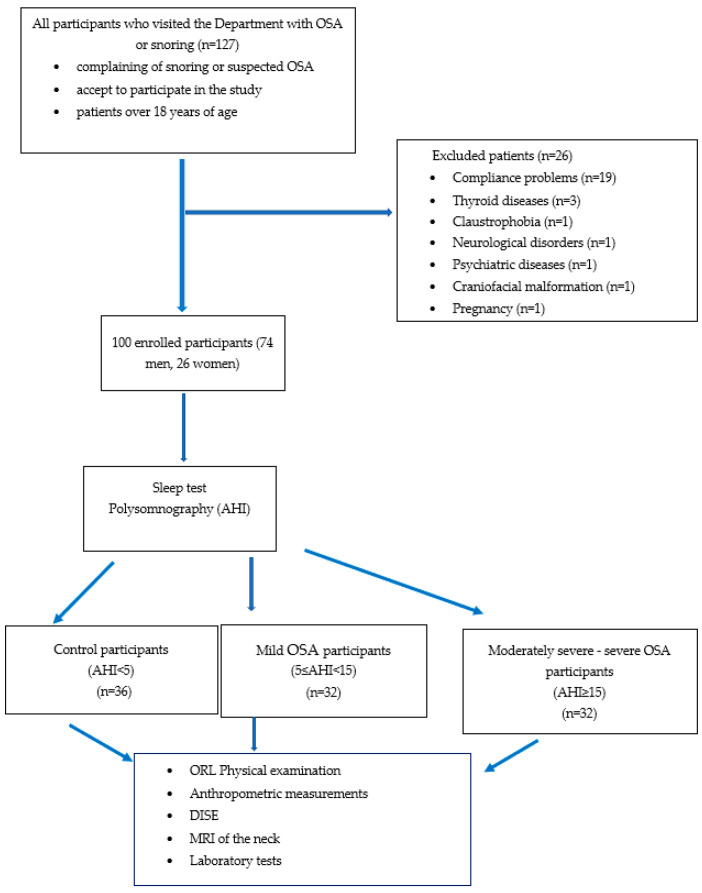
Study participants’ flow chart. AHI = apnoea-hypopnoea index, DISE = drug-induced sleep endoscopy, MRI = magnetic resonance imaging, OSA = obstructive sleep apnoea, ORL = otorhinolaryngological.

This prospective investigation was conducted at the Department of Otolaryngology and Head and Neck Surgery of Semmelweis University, and included one hundred participants (74 men and 26 women, mean age ± SD, 42.15 ± 11.7 years). Those over 18 years of age with snoring or suspected OSA, who gave their consent to participate in the investigation, were enrolled. Those who previously had oral or otorhinolaryngological surgeries, those who had craniofacial malformations (e.g., Down syndrome), had claustrophobia, soft tissue or thyroid gland disorders, neurological or psychiatric diseases, and those with alcohol or drug abuse or pregnancy were excluded. All participants were examined using a general otorhinolaryngological examination, a sleep test (i.e., polysomnography), drug-induced sleep endoscopy, and MRI of the neck region. The flow chart is presented in Figure 1.

The study was approved by the Hungarian Research Ethics Authority (National Institute of Pharmacy and Nutrition, approval reference number: 2788/2019). All patients gave their informed consent in writing.

Figure 1 shows the study population’s flow chart.

### 2.2. Anthropometric Measurements

Participants’ general anthropometric parameters, such as gender, age, body height, weight, and BMI, were calculated. Neck circumference was measured in the cricothyroid membrane, hip circumference in the anterior superior iliac spine, and abdominal circumference in the umbilicus, using a tape measure in each case.

### 2.3. Sleep Test

A SOMNOscreen Plus PSG device (SOMNOmedics GMBH Germany) was applied for overnight polysomnography, at the Institute of Pulmonology Törökbálint, under medical supervision. The examination results were adapted according to the American Academy of Sleep Medicine. Apnoea is determined as a reduction of 90% or more airflow through the oronasal thermistor for 10 s or more and hypopnoea as a reduction of 30% or more airflow, accompanied by a desaturation or arousal of 3% or more oxyhaemoglobin. The severity of OSA can be classified according to the apnoea-hypopnoea index (AHI) [21]. Due to the relatively low number of participants, they were classified into control (AHI < 5), mild OSA (5 ≤ AHI < 15), and moderately severe–severe OSA (AHI ≥ 15) groups.

### 2.4. MRI

The MRI examinations were performed at the Medical Imaging Centre of Semmelweis University, using a Philips Ingenia 1.5 T MRI device. Neck MRI was conducted using coronal T1 TSE (with 3.5 mm slice thicknesses without a gap), axial T2 SPIR, T1 TSE, and DWI measurements (with 3 mm slice thicknesses with a 1.5 mm gap), and sagittal T2 TSE, STIR, and T1 TSE analyses. Examinations were performed from the posterior nasal spine to the hyoid bone. Participants were instructed to breathe normally through their nose and avoid movements and swallowing. The images were analysed by an experienced radiologist using a Philips IntelliSpace Portal (Philips Healthcare, Best, The Netherlands).

**Figure 2:**
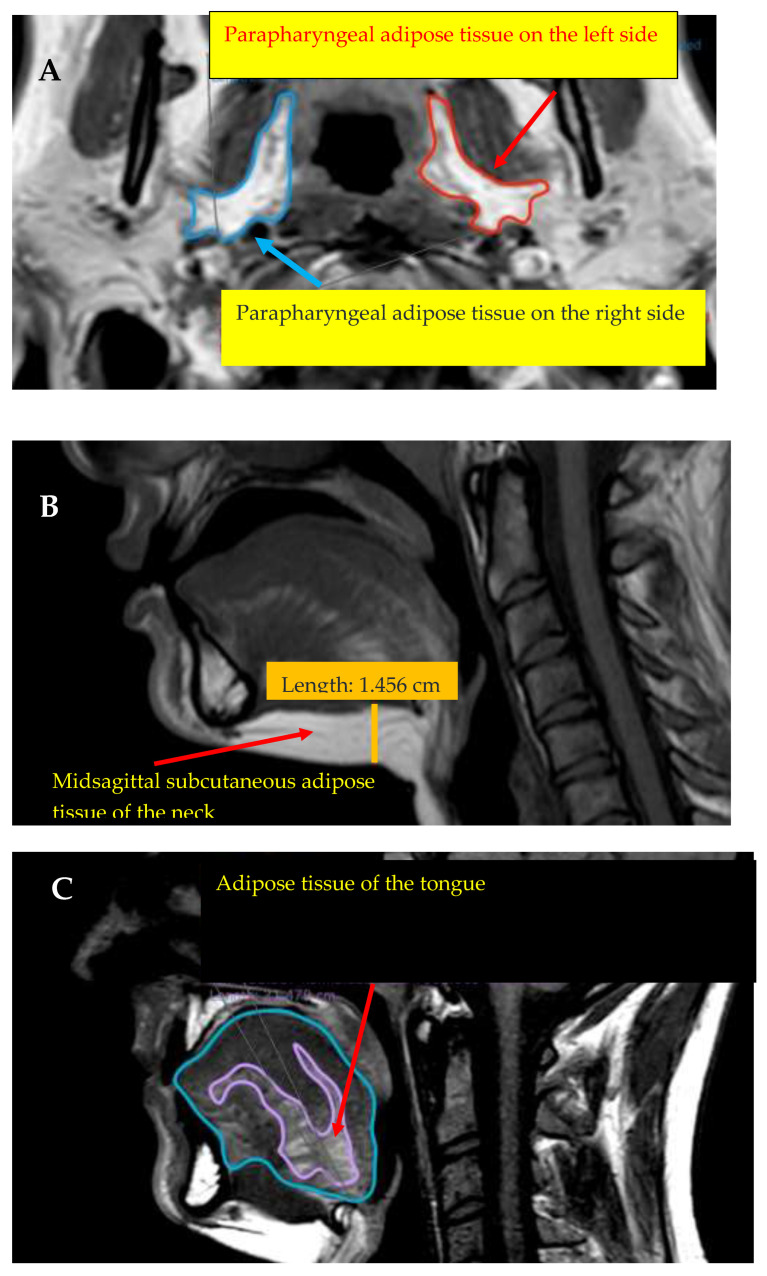
(**A**) T1-weighed measurements in the axial plane showing parapharyngeal adipose tissue on the left and right sides; (**B**,**C**) midsagittal axis of the T1-weighted MRI scans showing the midsagittal subcutaneous adipose tissue of the neck and adipose tissue of the tongue (taken from our data).

Parapharyngeal adipose tissue was defined as the largest extent of parapharyngeal fat tissue in the axial plane on both sides of the parapharyngeal wall and its areas were calculated using the region of interest (ROI) tool in the DICOM viewer. The thicknesses were also determined in the axial plane, using T1-weighed measurements using the ruler tool of the software. To calculate the estimated percentage of tongue fat, the area of the tongue in the midsagittal axis of the T1-weighted images with the ROI tool was also measured. In the next analysis step, the well-defined contiguous areas of the tongue adipose tissue in the same plane were differentiated using the same tool. Then, the tongue area and the areas of the tongue fat tissue were compared and a rough percentage of the ratio of the adipose tissue to the tongue was calculated from the measured data. The neck SAT was determined in the midsagittal region and was of the parapharyngeal AT in the axial plane, using T1-weighed measurements and the contour of the analysed region [22,23,24]. The MRI parameters are presented in Figure 2.

### 2.5. Drug-Induced Sleep Endoscopy

Drug-induced sleep endoscopy was performed in an operating room. A quantity of 1.5 mg per kilogram of propofol was applied for sedation and an Olympus flexible endoscope was inserted through the nose to the larynx. The results were adapted according to the VOTE classification, making it possible to determine precisely the location, severity, and configuration of the obstruction. Consequently, location could be determined as ‘V’, velum; ‘O’, oropharynx; ‘T’, tongue base; and ‘E’, epiglottis. The severity of obstruction could be 0, indicating that there is no obstruction; ‘1’, indicating a partial obstruction; or ‘2’, a total obstruction. ‘X’ means that the obstruction cannot be visualised. Configuration ‘L’ means a lateral, ‘AP’ an anteroposterior, and ‘C’ a concentric type of obstruction [25].

### 2.6. Data Processing

The correlations were examined using linear regression analysis. The differences between the grouping criteria were examined using the one-way analysis of variance (ANOVA). A critical condition of ANOVA is the homogeneity of variances, which was tested by Levene’s test. The test values were above the critical level of 0.05; therefore, the homogeneity of variance criterion was satisfied. Based on this, the differences between groups were analysed by the Bonferroni test. This is the most widely used test for multiple comparisons, and is capable of detecting differences similar to the relatively conservative tests (e.g., Schaffé’s S test) [26].

Our original idea was to determine the relationships between AHI and different parameters of the anthropometric and adipose tissue by a multivariable regression equation. However, the correlations between the parameters of the adipose tissue (i.e., independent variable) and the AHI values (i.e., dependent variable) were seen to be weak. This can be explained by the relatively high variance of the independent variables. Similar results were also obtained using the linear quadratic discriminant analysis. Consequently, more robust methods were selected which are less sensitive to the variance of the input variables. Therefore, each input variable was classified into three equal groups. In this case, the traditional logistic regression method was unable to be applied, as the number of empty cells was more than two-thirds of the total cells. At the same time, the unpredictable development of artificial intelligence, generally, and specifically machine learning, offers new tools for automatic, supervised patients’ classification in the case of AHI and obstructions based on their demographic, anthropometric, and adipose tissue parameters. Different classification algorithms have been tested, indicating an abundance of methods [27]. The efficiency of the ‘classic’ machine learning algorithms (e.g., random forest method) was found to be relatively poor. Therefore, the methods and algorithms developed for classification problems in chemometrics were selected because, in this field, the number of independent variables (inputs) is generally relatively high, compared to the number of samples (records) [28]. Consequently, it was possible to find algorithms to classify patients according to input parameters with unexpected efficiency. The most favourable results were produced using the Flexible Discriminance analysis and the Multivariate Adaptive Regression Splines. The algorithms can be found in the freely downloadable ‘mda’ R-package [29]. Garson’s method was used to test the relative significance of each parameter [30]. The cross-validation index was applied to detect the location of the obstruction [31].

The present investigation, such as all academic endeavours, had to combine the ambition to achieve well-founded results with the restriction of limited resources. Based on our preliminary calculations, following the recommendations of Shuster, an ideal sample should be three–five times larger [32]. This is a considerable difference, but we did not achieve one order of magnitude. The relatively low but not extraordinarily small sample size is an inherent limitation of the generalisability of results, although modern statistical methods, mainly bootstrapping, offer a favourable possibility to evaluate the robustness of results [33]. However, the application of cross-validation methods in sampling in the case of classifications considerably contributes to increasing reliability. Notwithstanding this, it should be noted that our results can only be considered to be preliminary. Validity must be further analysed and improved by increasing patients’ numbers and involving other races with different craniofacial and obesity characteristics.

## 3. Results

Of the 100 participants, 36 belonged to the control, and 32 to the mild and 32 to the moderately severe–severe OSA group. A male predominance was observed in all groups. Additionally, a higher ratio of participants under 40 years of age and a lower BMI was detected in the control group. Patients over 40 years of age and with higher BMI values were found in the OSA groups.

### 3.1. Basic Demographic Values, Laboratory Test, and AT MRI Parameters

The groups’ basic demographic values, laboratory test parameters, and AT MRI parameters are summarised in Table 1.

As Table 1 reveals, in the case of the anthropometric and most MRI parameters, a significant difference was observed between the OSA and control groups. Of the parameters examined, only the values of tongue fat%, total cholesterol, and LDL-cholesterol did not differ between the groups.

### 3.2. Correspondence between Demographic and MRI Parameters along with AHI and BMI

The correspondence between demographic and MRI parameters, along with AHI and BMI, is presented in Table 2.

As Table 2 reveals, most anthropometric and BMI parameters were correlated with BMI, of which the correlations with abdominal and hip circumferences and midsagittal neck SAT were the strongest. However, no significant correlations with AHI were observed.

### 3.3. Prediction of Velopharyngeal Obstruction

The prediction of velopharyngeal obstruction using anthropometric and MRI parameters is summarised in Table 3 and Table 4.

As Table 3 and Table 4. present, in the case of both of the above-mentioned statistical methods, the most vital parameters were the parapharyngeal AT, followed by the circumference and age. Velopharyngeal obstruction could be predicted in 80% of the cases using these parameters. In the other 16% of the cases, the algorithm incorrectly predicted velopharyngeal obstruction, and, in 5%, the algorithm did not detect the presence of obstruction.

### 3.4. Prediction of Oropharyngeal Obstruction

The correlation between oropharyngeal obstruction, and anthropometric and MRI parameters and their predictive values, is presented in Table 5 and Table 6.

As can be seen from Table 5, the prediction of oropharyngeal obstruction was efficient in 77% of cases using anthropometric and MRI parameters. In the other 14% of cases, the algorithm indicated false obstruction, and in 9%, it was unable to predict the presence of obstruction.

The relative importance of different parameters in predicting obstruction is summarised in Table 6.

Table 6 shows that BMI played the most crucial role in the prediction, although abdominal circumference and the sum of parapharyngeal AT also contained important information. The relatively low confidence interval indicates that the possibility of an obstruction in the case of higher BMI values is relatively high. In the case of lower BMI values, the sum of the parapharyngeal AT parameter is essential to predict obstruction.

### 3.5. Prediction of Tongue-Based Obstruction

The prediction of tongue-based obstruction applying anthropometric and MRI parameters is summarised in Table 7.

As shown in Table 7, the tongue-based obstruction could be predicted in 79% of cases, using anthropometric and MRI parameters. However, the algorithm indicated a false obstruction in 17% of cases and a false negative obstruction in 4% of cases.

### 3.6. Prediction of OSA

The prediction of OSA, applying anthropometric, laboratory test, and MRI parameters, and the relative significance of each parameter, were analysed using artificial intelligence (i.e., the Garson test). The efficiency of OSA categorisation is presented in Table 8, while the relative significance of each parameter is presented in Table 9.

Including anthropometric, laboratory test, and MRI parameters, using artificial intelligence, the presence of OSA could be predicted in 99% of cases. This means that the algorithm performed a false calculation in only one non-OSA case. To validate the results, the data were randomly divided into ’teaching’ and ’test’ parts in a 75:25 ratio. After hundreds of analyses, the average OSA prediction was over 90%. Age, tongue fat%, and neck circumference were determined as the essential parameters in the prediction, followed by laboratory test parameters (i.e., triglyceride and HDL-cholesterol). Left-sided parapharyngeal AT was also essential in the prediction, preceding other parameters, such as BMI and hip circumference.

## 4. Discussion

OSA affects a significant proportion of society and the ratio of undiagnosed cases is high; therefore, its diagnosis must be improved. Although many possibilities regarding the diagnosis of OSA are accessible, the earliest diagnosis is essential, due to the appearance of comorbidities. The primary purpose of diagnostic methods is to easily and quickly screen for OSA or diagnose the disorder with high specificity and sensitivity. Efficient screening is possible using self-administered questionnaires, of which the STOP-BANG (i.e., snoring, tiredness, observed apnoea, high blood pressure, BMI, age, neck circumference, and male gender) is generally used with reliable results. This questionnaire contains eight questions, and patients can answer with ‘yes’ or ‘no’ [34]. In addition, screening is also possible using the Berlin, Epworth, or STOP questionnaires. A meta-analysis that included 108 investigations with 47,989 participants determined a significantly higher sensitivity of the STOP-BANG questionnaire, although its specificity was lower than that of the Epworth questionnaire [35]. An alternative diagnostic approach is a home-sleep test (HST, Types III or IV), which can be effectively used when there is a high risk of moderate or severe OSA. The one-night polysomnography, in which sleep specialists interpret the results, is essential in the follow-up of the effectiveness of therapy and the diagnosis of OSA [36]. Notwithstanding the relatively low specificity of the questionnaires and their time requirement, and the necessity for qualified staff for home sleep tests, alternative methods, e.g., using artificial intelligence, are necessary. Previous results indicated that anthropometric parameters, the Epworth questionnaire, and expired gas analysis using machine learning could effectively predict OSA; only in 5.7% was a false mild instead of severe classification found [13]. The prediction of OSA based on machine learning was improved when the model was completed with physical examination parameters [14]. The prediction based on anthropometric and craniofacial parameters and the STOP-BANG questionnaire was more efficient in cases of moderate to severe OSA with no daytime symptoms [15]. Based on the correspondences mentioned above, the examination of vital OSA risk factors is not only essential regarding OSA pathophysiology, but using modern statistical methods (e.g., artificial intelligence), their role in OSA prediction can also be analysed.

The correlation between OSA and obesity is highly complex and has been particularly investigated; however, there are still some questions remaining. The present study aimed to investigate the role of anthropometric and AT MRI parameters of the neck, tongue, and parapharyngeal regions in the pathogenesis and prediction of OSA, plus the obstruction and location of the upper airways. Determining the correlation between BMI and AT is relatively easy; however, the correspondence between OSA and AT is more complex, as these correlations are not intuitive and cannot be described using simple functions. Therefore, other methods must be applied to analyse the correspondence between AT and anthropometric parameters (i.e., independent variables) and OSA (i.e., dependent variable) and predictive values. Consequently, artificial intelligence (i.e., Flexible Discriminance analysis and Multivariate Adaptive Regression Splines) was applied in our analyses.

The significance of the present investigation is that the use of artificial intelligence in OSA diagnostics on a relatively large sample was analysed.

Based on the fact that obesity is one of the most critical risk factors for OSA and is also correlated depending on age and sex with the severity of OSA, including anthropometric and AT MRI parameters of the upper airways, the severity categories of OSA could be correctly determined in 99% of cases. In the prediction, gender and hip circumference showed the most vital role. Carlisle et al. also observed the effect of age on pharyngeal morphology. In the case of older males, a higher retropharyngeal and retroglossal length was observed, along with the cross-sectional area of the soft palate and the diameter and cross-sectional area of the parapharyngeal fat pad, in that study [37,38]. The effect of age is also presented in increased genioglossus muscle activity in older awake males [39], which decreases during sleep, leading to vulnerability and collapsibility of the upper airways [40]. In the OSA prediction, age was a key factor based on the results of the current investigation, although no significant correlation with AHI and BMI was observed. Neck circumference was defined as the second essential parameter in the OSA prediction of anthropometric parameters. The literature contains conflicting data on neck circumference in OSA; some investigations have concluded a strong correlation between neck circumference and OSA severity [41,42], while others have not [43,44]. A meta-analysis regarding the correlation between neck circumference and obesity stated a sensitivity of neck circumference in the prediction of obesity of 80% and a specificity of 85% [45]. Neck circumference was defined to correlate with age, BMI, and hip and waist circumference, in both men and women [46].

In the OSA prediction, the algorithm indicated the anthropometric and left-sided parapharyngeal and tongue fat midsagittal MRI parameters as being the most crucial, showing a strong correlation with obesity. Accumulation of AT near the upper airways (i.e., tongue, parapharyngeal space, and central region) in obesity leads to increased collapsibility of the pharynx by mechanical effects and based on neuromuscular regulations in the central nervous system [47]. Compared to other somatic muscles, AT accumulation in the tongue showed a higher correlation with BMI and therefore, with obesity severity, which is strongly correlated with OSA severity [48]. Jugé et al. found similar results, and observed a significant positive correlation between tongue AT and BMI and older ages [49]. Our results showed that tongue fat% did not significantly differ between OSA categories and the control group, but the midsagittal region fat% parameter did. The tongue fat% neither correlated with AHI nor BMI; however, tongue midsagittal region fat% significantly correlated with BMI. Kim et al. observed a significant positive correlation between tongue fat volumes and AHI and BMI. Furthermore, a higher percentage of tongue fat% in the OSA group was detected; however, there was no significant difference compared to the control group [50]. Parapharyngeal AT is strongly correlated with obesity, highlighted by the correlation between parapharyngeal AT and BMI. However, no correlation with AHI was detected. Consequently, parapharyngeal AT parameters contained essential information in the algorithm; however, they did not significantly correlate with OSA severity. Chen et al. detected a significant correlation between AHI and the subglosso-supraglottic-level parapharyngeal fat pad, independently of BMI and neck circumference parameters [51]. According to Gao et al., in patients with a BMI over 28 kg/m^2^, a significant positive effect of age on parapharyngeal AT volumes was detected [52].

In predicting velopharyngeal obstruction, the algorithm determined parapharyngeal AT as the most vital parameter, followed by neck circumference and age. Using these parameters, by artificial intelligence, the velopharyngeal obstruction could be correctly detected in 80% of cases. Jang et al. detected a higher percentage of retropalatal concentric obstruction in patients with OSA with higher parapharyngeal AT volumes [53]. This is in agreement with our results, referring to the significant role of parapharyngeal AT in velopharyngeal obstruction.

The importance of parapharyngeal AT in predicting oropharyngeal obstruction was not found, in contrast to the prediction of velopharyngeal obstruction, since BMI was indicated as the most crucial parameter, followed by abdominal circumference and parapharyngeal AT, with the latter two showing the same importance. Applying these three parameters, using artificial intelligence, the oropharyngeal obstruction could be predicted in 77% of cases. However, interestingly, the algorithm did not determine the other anthropometric and MRI parameters that are essential for prediction. Pahkala et al. highlighted the importance of increased lateral pharyngeal collapsibility associated with accumulation of parapharyngeal adipose tissue in obese patients, explained by impaired mechanisms controlling passive collapse of the pharyngeal wall [54]. However, Li et al. determined the increased mechanical loading of parapharyngeal AT on the lateral pharyngeal wall as a possible background [22]. Chen et al. observed a strong correlation between subglosso-supraglottic-level AT and lateral pharyngeal obstruction at the same level [51].

Regarding tongue-based obstruction, the higher tongue volume, the adipose deposits accumulated in the tongue, and the decreased muscle activity during sleep can be defined, and are also negatively influenced by the accumulated intramuscular AT [55]. To predict tongue-based obstruction, BMI was defined as the most vital parameter; thus, tongue-based obstruction could be predicted in 79% of cases. The correspondence between BMI and tongue volumes is highly complex; some researchers have indicated a strong correlation between them [56], while others have not [50]. According to our investigation results, tongue volumes significantly correlated with AHI and BMI in both sexes, while tongue fat significantly correlated with BMI.

Finally, it can be concluded that an MRI of the adipose tissue surrounding the upper airways can be an alternative examination of OSA when an MRI in the neck region is used with another indication other than OSA. Both OSA and velopharyngeal obstruction can be predicted using artificial intelligence. Compared to self-administered questionnaires, an essential advantage of our algorithm is that the location of obstruction can be identified with high precision and the examination is relatively fast compared to the home sleep test. Our results are especially crucial in cases where MRI was performed and was previously not diagnosed.

The present investigation had some limitations. First, the relatively low number of participants did not allow for the division of OSA into categories based on its severity. Moreover, the magnetic resonance examinations were performed on awake subjects and, therefore, did not present the situations during physiological sleep.

## 5. Conclusions

Based on the results of the present investigation, the MRI-based AT and anthropometric parameters were not significantly correlated with OSA severity; however, a significant correlation with BMI was detected. Parapharyngeal AT plays a significant role in the presence of velopharyngeal obstruction and OSA pathophysiology. However, it has a limited effect on oropharyngeal obstruction; moreover, it does not affect tongue-based obstruction. The BMI was defined as the most vital parameter of oropharyngeal and tongue-based obstruction; furthermore, its role in OSA pathophysiology is also significant. Neck circumference is essential to predict velopharyngeal obstruction and OSA, and abdominal circumference to predict oropharyngeal obstruction and OSA. In predicting OSA, age was determined as the most vital parameter, followed by the tongue fat midsagittal and neck circumference parameters, which had the same importance. In conclusion, using anthropometric and MRI AT parameters, by artificial intelligence, OSA and upper-airway obstruction can be predicted in 99% of cases, velopharyngeal obstruction in 80%, oropharyngeal obstruction in 77%, and tongue-based obstruction in 79%.

## Figures and Tables

**Table 1:** Patients’ basic demographic, MRI, and laboratory test results. The parameters show the mean ± SD values. *** indicates the significant difference at *p* < 0.01 level, while ** the significant difference at *p* < 0.05 level and * the significant differences at *p* < 0.1.

Indicators	Control Group *n* = 36 (A)	Mild OSA *n* = 32 (B)	Moderately Severe + Severe OSA *n* = 32 (C)	*p*-Value	Differences
Age (years)	38.42 ± 12.13	45.34 ± 11.17	43.16 ± 10.9	0.042 **	A-B
Weight (kg)	78.94 ± 13.15	93.03 ± 14.58	101.97 ± 17.21	0.000 ***	A-B; A-C; B-C
Hip circumference (cm)	100.49 ± 11.97	106.28 ± 10.46	111.15 ± 10.96	0.001 ***	A-C
Abdominal circumference (cm)	94.73 ± 12.7	104.97 ± 11.68	111.01 ± 12.51	0.000 ***	A-B; A-C
Neck circumference (cm)	37.95 ± 4.12	40.69 ± 3.42	42.73 ± 3.33	0.000 ***	A-B; A-C; B-C
Tongue fat midsagittal (cm^2^)	824.82 ± 159.43	928.64 ± 154.39	933.66 ± 176.27	0.01**	A-B; A-C
Tongue fat (%)	0.33 ± 0.05	0.33 ± 0.05	0.33 ± 0.06	0.957	No significant difference
Midsagittal SAT of the neck (mm)	6.1 ± 1.69	6.62 ± 1.75	7.26 ± 1.57	0.019 **	A-C
Parapharyngeal AT on the right side (cm^2^)	253.47 ± 62.88	269.89 ± 64.37	304.58 ± 61.64	0.004 **	A-C; B-C
Parapharyngeal AT on the left side (cm^2^)	256.19 ± 63.67	285.45 ± 83.97	311.63 ± 60.54	0.006 **	A-C
Sum of the parapharyngeal AT (cm^2^)	509.66 ± 121.96	555.34 ± 141.88	616.21 ± 110.07	0.003 **	A-C
Total cholesterol (mmol/L)	5.59 ± 1.15	5.9 ± 1.17	5.47 ± 1.01	0.279	No significant difference
HDL-cholesterol (mmol/L)	1.31 ± 0.29	1.22 ± 0.32	1.13 ± 0.17	0.024 **	A-C
LDL-cholesterol (mmol/L)	3.58 ± 0.81	3.89 ± 0.82	3.7 ± 0.8	0.282	No significant difference
Triglycerides (mmol/L)	1.82 ± 1.33	2.58 ± 1.8	2.04 ± 1.17	0.097 *	A-B

**Table 2:** Pearson’s correlation coefficients (r^2^) between basic demographic values and MRI, and AHI and BMI. The parameters show the correlation coefficients. ** indicates the significant difference at *p* < 0.05 level.

	Correlation with	
Indicators	BMI (kg/m^2^)	AHI (events/hour)
	*p*-Value	*p*-Value
Age (years)	0.015	0.066
Hip circumference (cm)	0.793 **	0.011
Abdominal circumference (cm)	0.872 **	−0.014
Neck circumference (cm)	0.357 **	−0.035
Tongue fat midsagittal (cm^2^)	0.358 **	−0.035
Tongue fat (%)	0.146	0.022
Midsagittal SAT of the neck (mm)	0.509 **	−0.167
Parapharyngeal AT on the right side (cm^2^)	0.311 **	−0.067
Parapharyngeal AT on the left side (cm^2^)	0.299 **	−0.125
Sum of the parapharyngeal AT (cm^2^)	0.322 **	−0.103

**Table 3:** Prediction of velopharyngeal obstruction using anthropometric and MRI parameters. The table presents the categorisation of real and predicted velopharyngeal obstruction, along with number of patients in each group.

		Reference	
		Non-Velopharyngeal Obstruction	Velopharyngeal Obstruction
Prediction	Non-Velopharyngeal Obstruction	16	5
	Velopharyngeal Obstruction	15	64

**Table 4:** Relative significances of the parameters in the prediction of velopharyngeal obstruction, applying two statistical approaches (i.e., general cross-validation and residual sum-square methods). The table shows the role of different factors in predicting velopharyngeal obstruction, indicating the relative importance of each parameter.

Number of Subsets	General Cross Validation	Residual Sum Squares
Sum of the parapharyngeal AT (cm^2^)	100.0	100.0
Neck circumference (cm)	53.8	65.9
Age (years)	30.7	47.1

**Table 5:** Prediction of oropharyngeal obstruction using anthropometric and MRI parameters. The table presents the categorisation of real and predicted oropharyngeal obstruction, together with patient numbers in each group.

		Reference	
		Non-Oropharyngeal Obstruction	Oropharyngeal Obstruction
Prediction	Non-Oropharyngeal obstruction	20	9
	Oropharyngeal obstruction	14	57

**Table 6:** Prediction of oropharyngeal obstruction using anthropometric and MRI parameters, by general cross-validation index. The table shows the role of the different parameters in predicting oropharyngeal obstruction in%, indicating the relative importance of each parameter.

Indicators	General Cross-Validation Index Value	Residual Sum of Squares
BMI (kg/m^2^)	100.0	100.0
Sum of the parapharyngeal AT (cm^2^)	44.1	68.4
Abdominal circumference (cm)	44.7	57.2

**Table 7:** Prediction of tongue-based obstruction, including anthropometric and MRI parameters. The table presents the 100 patients’ real and predicted categorisation of obstruction along with patient numbers in each group.

		Reference	
		Non-Tongue-Based Obstruction	Tongue-Based Obstruction
Prediction	Non-Tongue-based obstruction	12	4
	Tongue-based obstruction	17	67

**Table 8:** Prediction of OSA categories, including anthropometric, laboratory test, and MRI parameters. The table presents 100 patients’ real and predicted OSA categorisation, along with patient numbers in each group.

Estimated	OSA Categories		
	Control	Mild OSA	Moderately Severe + Severe OSA
Non-OSA	34		
Mild OSA	1	33	
Moderately severe + Severe OSA			32

**Table 9:** Relative importance of different factors (%) by multivariate discriminant analysis in the prediction of OSA subcategories, including anthropometric, laboratory test, and MRI parameters. The table shows the role of the different parameters in predicting OSA in%, indicating the relative importance of each parameter.

Indicators	Importance (%)
Age (years)	10.8
Tongue fat midsagittal (cm^2^)	7.8
Neck circumference (cm)	7.7
Triglycerides (mmol/L)	7.5
HDL-cholesterol (mmol/L)	7.3
Parapharyngeal AT on the left side (cm^2^)	7.1
Total cholesterol (mmol/L)	6.8
Hip circumference (cm)	6.1
BMI (kg/m^2^)	5.65
Weight (kg)	4.9
Height (cm)	4.75
Abdominal circumference (cm)	4.5
LDL-cholesterol (mmol/L)	4.4
Tongue fat %	4.1
Parapharyngeal AT on the right side (cm^2^)	3.8
Gender	3.6
Midsagittal SAT of the neck (mm)	3.2

## Data Availability

Data supporting reported results can be provided upon reasonable request.
